# Association between alexithymia and substance use: A systematic review and meta‐analysis

**DOI:** 10.1111/sjop.12821

**Published:** 2022-04-18

**Authors:** Kirsi Honkalampi, Markus Jokela, Soili M. Lehto, Mika Kivimäki, Marianna Virtanen

**Affiliations:** ^1^ School of Educational Sciences and Psychology University of Eastern Finland Joensuu Finland; ^2^ Department of Psychology and Logopedics University of Helsinki Helsinki Finland; ^3^ Institute of Clinical Medicine University of Oslo Oslo Norway; ^4^ R&D department, Division of Mental Health Services Akershus University Hospital Lørenskog Norway; ^5^ Department of Psychiatry University of Helsinki Helsinki Finland; ^6^ Clinicum, Faculty of Medicine University of Helsinki Helsinki Finland; ^7^ Department of Epidemiology and Public Health University College London London UK; ^8^ Division of Insurance Medicine Karolinska Institutet Stockholm Sweden

**Keywords:** Alexithymia, alcohol, drinking behavior, drugs, meta‐analysis, substance use, TAS‐20

## Abstract

Alexithymia has been associated with substance use, but the magnitude of the association has not been evaluated and sub‐group differences, if any, are unknown. The aim of this meta‐analysis is to systematically review the association between alexithymia and substance use (alcohol or illicit drugs). We identified studies through a systematic review of PubMed and Web of Science and obtained a total of 52 publications using the Toronto Alexithymia Scale‐20 scale. Random effects meta‐analysis was used to evaluate the overall and sub‐group associations. Of the studies, 50 were cross‐sectional and two longitudinal. Alexithymia was associated with any substance use (Cohen’s *d* = 0.62, 95% confidence interval [CI] 0.49–0.76), with little difference between estimates for use of alcohol or illicit drugs. A stronger association was observed for the alexithymia dimension “Difficulty in Identifying Feelings” (*d* = 0.64, 95% CI = 0.47–0.81) and “Difficulty in Describing Feelings” (*d* = 0.44, 95% CI = 0.32–0.55) than for “Externally Oriented Thinking” (*d* = 0.19, 95% CI = 0.09–0.28). The association was stronger in studies with clinical patient populations (*d* = 0.83, 95% CI = 0.62–1.05) than in those investigating general or student populations, and in studies with a majority of male rather than female participants. These findings suggest a strong overall association between alexithymia and substance use and a very strong association among clinical patient populations. The association may be stronger with the emotion‐related dimensions than with the cognition‐related dimension of alexithymia. As nearly all the studies were cross‐sectional, more longitudinal studies are needed.

## INTRODUCTION

Alexithymia was originally defined as the inability to recognize and verbalize emotions (Sifneos, 1973). A poverty of imagination or of a fantasy world as well as a lack of positive emotions and a high prevalence of negative emotions have also been described as characteristic of alexithymia (Taylor, 2018). Alexithymia is often viewed as a personality trait with a normal distribution among the population (Bagby, Parker & Taylor, 1994). The most commonly used scale for assessing alexithymia is the self‐reported Toronto Alexithymia Scale with 20 items (TAS‐20) (Bagby *et al*., 1994; Parker, Taylor & Bagby, 2003; Taylor, Bagby & Parker, 2003). TAS‐20 scale includess: Difficulty in Identifying Feelings (DIF), Difficulty in Describing Feelings to others (DDF), and Externally Oriented Thinking (EOT). The replicability of the three‐factor structure of TAS‐20 has been demonstrated in both clinical and non‐clinical populations and TAS‐20 has been translated into over twenty languages (Bressi, Taylor, Parker *et al*., 1996; Joukamaa, Miettunen, Kokkonen *et al*., 2001; Luminet, Olivier, Taylor & Bagby, 2018).

Substance use disorder (SUD) as a diagnosis in the DSM‐IV is a condition in which there is uncontrolled use of a substance (i.e., alcohol, cocaine, heroin, opioid, sedatives, or stimulants) despite harmful consequence (Livne, Shmulewitz, Stohl, Mannes, Aharonovich & Hasin, 2021). The alcohol abuse and other addiction problems has been shown to be associated with alexithymia (Thorberg, Young, Sullivan & Lyvers, 2009). The earliest studies found a high prevalence of alexithymia (40–50%) among patients diagnosed with alcohol abuse or dependence (Thorberg *et al*., 2009; Uzun, Ates, Cansever & Ozsahin, 2003).

In addition, several studies have reported a high prevalence of alexithymia among subjects with illicit drug abuse, El Raasheed (2001) showed that alexithymic heroin addict individuals reported more polysubstance abuse, more opiate use (other than heroin) and more benzodiazepine abuse than non‐alexihymic individuals. In addition, Payer, Lieberman and London (2011), and Hamidi, Rostami, Farhoodi and Abdolmanafi (2010) have reported higher levels of alexithymia in heroin dependent individuals compared to the healthy control group. Interestingly, Bulai and Enea (2016) compared three addictive groups (tobacco, cannabis, and alcohol) and found that the alcohol abusers have a significantly higher level of alexithymia than cannabis abusers, smokers, and controls. In addition, Ghalehban and Besharat (2011) found that patients with substance abuse disorder (without specifying the drug) score significantly higher TAS‐20 scores than normal individuals. Moreover, Patwardhan, Mason, Chmelka, Savolainen, Miettunen & Jarvelin (2019) found that none of the alexithymia domains was directly associated with substance use disorder (without specifying what drug the subjects have used) in adulthood among the Northern Finland Birth Cohort 1986, thus more information is needed to find out whether alexithymia is a trait which predisposes to alcohol or substance abuse.

Since then, two systematic literature reviews, and one meta‐analysis to date, have evaluated the relationship between alexithymia and alcohol use disorders (Pigoni, Mandolini, Delvecchio, Bressi, Soares & Brambilla, 2020; Thorberg *et al*., 2009). A recent meta‐analysis by Pigoni *et al*. (2020) evaluated the associations between non‐suicidal self‐injury (NSSI), risky drinking and alexithymia. They found 33 risky drink‐related articles. The results showed a significant positive association between TAS‐20 scores, DIF and DDF both NSSI and risky drinking. This association was stronger among ≥30 years of age. In addition, they found that EOT associated with risky drinking but not NSSI.

Thorberg *et al*. (2009) investigated the relationship between alexithymia and alcohol use disorder among alcohol‐dependent populations. They included 24 studies published between 1973 and 2008 in their systematic review and showed a higher prevalence rate of alexithymia in alcohol‐dependent populations compared with the control groups, and a positive relationship between alexithymia, The amount of alcohol use and severity of alcohol problems among the patient groups. Another systematic review, by Cruise and Becarra (2018), summarized findings from 30 studies published between 2009 and 2016. They found that alexithymia is an independent risk factor for alcohol‐related problems among clinical samples. Neither of the two systematic reviews performed a meta‐analysis to quantify the association between alexithymia and alcohol or drug use.

The aim of this study was to conduct a systematic review and meta‐analysis to update and further summarize the findings on the relationship between alexithymia and alcohol abuse and find out whether there are similar associations between alexithymia and drug use. In particular, we were interested in exploring whether some subtypes of alexithymia would be more strongly associated with abuse of alcohol or drugs than others.

We focus also on mediating variables. These included sample type (clinical, general etc. populations), age and sex distribution of the participants, and study region. We hypothesize that alexithymia is associated positively with alcohol and drug abuse and more strongly with DIF and DDF than EOT. We hypothesize that alexithymia is associated with both alcohol and drug abuse Furthermore, based on earlies findings (Pigoni *et al*., 2020; Thorberg *et al*., 2009), we hypothesize that alexithymia is associated with alcohol use and more strongly with alexithymia subtypes DIF and DDF rather than EOT. Furthermore, we hypotheses that drug use associated similarly to alexithymia as alcohol use. In addition, we hypotheses that this association is more common in clinical than population samples, and more common in males than in females. We hypothesize that participant's age (> 30 years) influences findings similarly as in Pigoni's *et al*. (2020) study.

## METHODS

We followed the Prisma guidelines for systematic reviews and meta‐analyses (http://Prisma‐statement.org/PRISMAStatement/FlowDiagram).

### Identification of the studies

We searched the PubMed and Web of Science databases for available publications from January 2000 to September (PubMed)/October (Web of Science) 2019. The search terms we used to identify suitable publications are shown in Supplementary Materials Table S1.

### Selection of studies

The study was included in the meta‐analysis if it: (1) was a cross‐sectional, case–control or cohort study including adult individuals (≥ 18 years) with alexithymia and alcohol/drug use assessments; (2) used TAS‐20 to assesses alexithymia; and (3) reported estimates for the association between alexithymia and substance use (e.g., Pearson correlation, mean difference, odds ratio (OR), relative risk (RR)). In addition, patient populations were accepted if the study included a “healthy” comparison group. The selected articles had to be written in English and be published in a peer‐reviewed journal. The resulting abstracts were read through and were included in the meta‐analysis if they fulfilled the inclusion criteria. We did not include other types of addiction than substance use, thus excluding, for example, internet or game addiction. When multiple reports on the same data had been published, we chose the study which included the most informative estimates or was published first. In addition to the database searches, we searched the reference lists of the eligible studies for additional records.

#### Data extraction

The description of the study population (patients or general population), the size of the study groups, the age of subjects, proportion of males, length of follow‐up and the method used for measuring alexithymia were extracted from each report. Two reviewers (KH, MV) independently checked the estimates derived in the studies on the association between alexithymia and substance use.

#### Quality assessment

Quality of the studies was assessed with a 14‐item instrument by the Quality Assessment Tool for Observational cohort and Cross‐sectional‐sectional studies, which belongs to the study quality assessment tools by NIH (National Heart, Lung, and Blood Institute; https://www.nhlbi.nih.gov/health‐topics/study‐quality‐assessment‐tools) (Table S2). Each of the dimensions of study quality was scored “0” if the study “does not include risk of bias” and “1” if the study “includes risk of bias,” “CD” if this was not possible to determine, “NA” if the item was not applicable,” and “NR” if the information was not reported. In principle, a study cannot be determined as having high quality if there is risk of bias rated to any of the items.

#### Meta‐analytic procedure

As there were several types of analyses in the original publications (mean difference, correlation and odds ratios), we transformed all original estimates to standardized mean differences (Cohen's *d*) and their 95% confidence intervals. We expected to find heterogeneity between studies and therefore used random‐effects meta‐analysis to obtain pooled estimates for the retrieved studies. The magnitude of Cohen's *d* was considered low if *d* ≤ 0.3, medium if *d* = 0.31 to 0.5, high if *d* = 0.51 to 0.8, and very high if *d* ≥ 0.81 (Cohen, 1988). Heterogeneity between studies was assessed by the *I* ^*2*^ test and meta‐regression was used to assess subgroup differences. Replanned subgroup analyses included the following: (1) outcome (DIF, DDF, EOT dimensions); (2) sample type (general, student, clinical); (3) mean age of the participants (<30 vs. ≥30 years); (4) proportion male (≤50% vs. >50%); (5) region (Europe, USA, Australia, Asia, Africa); (6) substance type (alcohol vs. other); and (7) analysis type (mean difference, correlation, logistic regression). We examined possible publication bias using the Egger's test for small‐study effects and a funnel plot of the estimates against their standard errors (Egger, Schneider & Davey Smith, 1998). Stata 13.1 (StataCorp, College Station, Texas, USA) was used for all meta‐analyses.

## RESULTS

### Searches

**Fig. 1:**
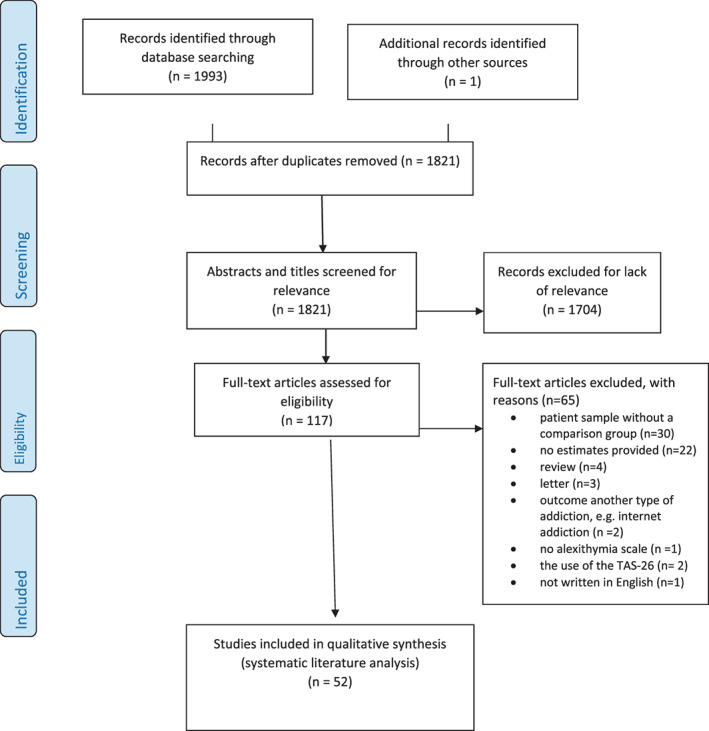
Flow diagram of the search stratyeg. [Colour figure can be viewed at wileyonlinelibrary.com]

The search process and its results are presented as a flow chart (Fig. 1). The searches identified a total of 1,993 documents to be screened, resulting in 1,821 after the removal of 172 duplicates. During the initial screening, the authors (KH and MV) independently assessed the titles and abstracts against the eligibility criteria. This screening resulted in a total of 117 records for a full text assessment. Any disagreements were discussed among the reviewers and resolved by consensus. The final dataset included 52 publications. One of the studies was found after manual searches of the reference lists of eligible studies (Zdankiewicz‐Ścigała & Ścigała, 2020). The reasons for the 65 exclusions were as follows: patient sample without a non‐patient comparison group (n = 30), review (n = 4) or letter (n = 3) without original data, another type of addiction, for example, internet addiction (n = 2), no effect estimates provided (n = 22), no alexithymia scale used (n = 1), the use of a modified version of TAS‐20 (e.g., TAS‐26, n = 2), and study not written in English (French, n = 1).

### Study populations

Descriptive statistics of the selected studies are presented in Table 1. In Table 1 the type of substance use has described in detail whenever it was possible. Several studies mentioned the diagnosed substance use disorder but did not specify the used substance (Bulai & Enea, 2016; Ghalehban & Besharat, 2011; Hamidi *et al*., 2010; Loas, Corcos, Stephan *et al*., 2001; Marchesi, Ossola, Tonna & De Panfilis, 2014; Parolin, Simonelli, Cristofalo *et al*., 2017; Patwardhan *et al*., 2019; Payer *et al*., 2011; Verrocchio, Conti & Fulcheri, 2010).

**Table 1:** Descriptive statistics of the 52 studies examining the association between alexithymia and substance use

Authors	Sample	Sample ‐type	N (controls)	Age	Male %	Country	The type of substance use	Adjusment
Andres *et al*. 2014	Student	Cross‐sectional	434	20	54	France	The amount of alcohol use	none
Bashapoor *et al*. 2015	Clinical	Case–control	36 (36)	30	100	Iran	SUD^a^	sex, gender, age, employment
Bauer & Ceballos, 2014	Student	Case control	42 (55)	19	0	USA	Alcohol binge drinking	sex, gender
Betka *et al*. 2018	General	Cross‐sectional	600	27	26	UK	Alcohol binge drinking	none
Bladt 2002	Student	Cross‐sectional	117 (122)	19	33 (28)	USA	Alcohol binge drinking	sex, gender
Bruce *et al*. 2012	Student	Cross‐sectional	862	26	24	UK	The amount of alcohol use	none
Bujarski *et al*. 2010	Student	Cross‐sectional	237	20	31	USA	The amount of alcohol use	none
Bulai & Enea 2016	Clinical	Case–control	131	26	72	Romania	Cannabis use Alcohol abuse	none
Chaudhury *et al*. 2006	Clinical	Case–control	100 (100)	38	100	India	AUD	age, sex, gender, region
Craparo *et al*. 2014	Clinical	Case–control	115 (117)	44	51	Italy	AUD	age, sex, gender
Craparo *et al*. 2016	Clinical	Case–control	31 (31)	34	81	Italy	AUD	age, sex, gender
Elander *et al*. 2014	General	Cross‐sectional	112	45	18	UK	Pain‐killers misuse	none
Founta *et al*. 2019	Clnical	Cross‐sectional	184	46	68	Ireland	AUD	none
Ghalehban & Beharat 2011	Clinical	Case–control	180 (180)	31	95	Iran	SUD^a^	age, sex, gender
Ghorbani *et al*. 2017	Clinical	Case–control	205 (100)	31	75	Iran	AUD	age, sex, gender
Gilan *et al*. 2015	Students	Cross‐sectional	250 (298)	22	42	Iran	Sedative use	none
Greene *et al*. 2020	Students	Cross‐sectional	183 (365)	22	23	Australia	Alcohol risky drinking	none
Hamidi *et al*. 2010	Clinical	Case–control	85 (85)	no	no	Iran	SUD^a^	age, sex, gender, SES
Hahn *et al*. 2016	Students	Cross‐sectional	425	19	29	USA	The amount of alcohol use	none
Hasking & Claes 2020	Students	Cross‐sectional	951	22	20	Australia	Risky use of alcohol	none
Honkalampi *et al*. 2010	General	Prospective	290	25–64	49	Finland	AUD	sex, gender, age, self‐reported health, work ability, depression
Knapton *et al*. 2018	General	Cross‐sectional	138	32	43	UK	The amount of alcohol use	none
Kopera *et al*. 2018	Clinical	Case–control	92 (86)	40	70	Poland	AUD	age, education, depression
Loas *et al*. 2000	Clinical	Case–control	60 (57)	36	80	France	AUD	age
Loas *et al*. 2001	Clinical	Case–control	659 (769)	27	41	France	SUD^a^	age, sex, gender
Lyvers *et al*. 2012a	General	Cross‐sectional	262	27	32	Australia	Alcohol risky drinking and alcohol use disorder	none
Lyvers *et al*. 2012b	General	Cross‐sectional	314	28	46	Australia	The amount of alcohol use	none
Lyvers *et al*. 2014a	Student	Cross‐sectional	113	22	31	Australia	The amount of alcohol use	none
Lyvers *et al*. 2014b	General	Cross‐sectional	100	21	28	Australia	The amount of alcohol use	none
Lyvers *et al*. 2014c	Clinical	Cross‐sectional	207	30	58	Australia	The amount of alcohol use	none
Lyvers *et al*. 2014d	Student	Cross‐sectional	153	21	43	Australia	SUD, 60% multiple users	none
Lyvers *et al*. 2018a	Student	Cross‐sectional	126	21	40	Australia	The amount of alcohol use	none
Lyvers *et al*. 2018b	General	Cross‐sectional	155	22	48	Australia	Alcohol risky drinking	none
Lyvers *et al*. 2018c	General	Cross‐sectional	161	23	41	Australia	The amount of alcohol use	none
Lyvers *et al*. 2019a	General	Cross‐sectional	224	27	18	Australia	The amount of alcohol use	none
Lyvers *et al*. 2019b	Student	Cross‐sectional	97	22	31	Australia	The amount of alcohol use	none
Lyvers *et al*. 2019c	General	Cross‐sectional	291	26	39	Australia	The amount of alcohol use	none
Lyvers *et al*. 2019d	General	Cross‐sectional	143	26	52	Australia	The amount of alcohol use	age, sex, gender, social desirability, personality
Marchesi *et al*. 2014	Clinical	Case–control	30 (78)	35	36	Italy	SUD (cocaine, heroin, multiple use)	age, sex, gender, education, anxiety, depression
Maurage *et al*. 2011	Clinical	Case–control	30 (30)	45	60	Belgium	AUD	age, sex, education
Maurage *et al*. 2017	Clinical	Case–control	296 (246)	48	72	Belgium	AUD	age, sex, gender
Meziou *et al*. 2019	Clinical	Case–control	50 (50)	35	100	Tunisia	Buprenorphine addiction	age, sex, gender, education
Parolin *et al*. 2017	Clinical	Case–control	41(27)	21	47	Italy	Polydrug users	age, sex, gender
Patwardhan *et al*. 2019	General	Longitude	6,186	28	49	Finland	SUD^a^	age, sex, gender
Payer *et al*. 2011	Clinical	Case–control	31(27)	32	55	Italy	Methamphetamine users	age, sex, gender
Pedersen *et al*. 2016	Physicians	Cross‐sectional	1841	30–60	50	Denmark	Risky use of alcohol	age, sex, gender
Rasheed 2001	Clinical	Case–control	200 (200)	27	100	Egypt	SUD^a^ (mostly opioids)	age, sex, gender, sociodemographic status
Shishido *et al*. 2013	Student	Cross‐sectional	429	20	31	USA	The amount of alcohol use	none
Speranza *et al*. 2004	Clinical	Case–control	208/123/controls	37	72/63	France	Alcohol/illicit drugs	age, sex, gender, SES
Verrocchio *et al*. 2010	Clinical	Case–control	77 (77)	29	100	Italy	Opioid's dependence	age, sex, gender
Zdankiewicz‐Ścigała & Ścigała, 2018	Clinical	Control Cross‐sectional	201	33	67	Poland	AUD	none
Zdankiewicz‐Ścigała & Ścigała, 2020	Clinical	Case–control	167 (90)	39	57	Poland	AUD	age

a SUD, Substance use disorder, but not specified any drugs; AUD, alcohol use disorder/dependence/addiction.

All publications reported cross‐sectional findings, with the exception of two Finnish studies (Honkalampi, Koivumaa‐Honkanen, Lehto *et al*., 2010; Patwardhan *et al*., 2019). A total of 22,712 individuals had participated in these studies, the number of participants in individual studies varying between 29 (Lyvers, Hayatbakhsh, Stalewski & Thorberg, 2019e) and 6,963 (Patwardhan *et al*., 2019). The majority, 23 studies, included clinical samples (Bulai & Enea, 2016; Chaudhury, Das & Ukil, 2006; Hamidi *et al*., 2010; Loas, Otmani, Lecercle & Jouvent, 2000; Loas *et al*., 2001; Lyvers, Hinton, Gotsis, Roddy, Edwards & Thorberg, 2014d; Meziou, Ghali, Khelifa *et al*., 2019; Parolin *et al*., 2017; Payer *et al*., 2011; Rasheed, 2001; Speranza, Corcos, Stephan *et al*., 2004; Verrocchio *et al*., 2010; Zdankiewicz‐Ścigała & Ścigała, 2018; Zdankiewicz‐Ścigała & Ścigała, 2020).

Thirteen investigations consisted of participants from the general population (Betka, Pfeifer, Garfinkel *et al*., 2018; Elander, Duarte, Maratos & Gilbert, 2014; Honkalampi *et al*., 2010; Knapton, Bruce & Williams, 2018; Lyvers,, Onuoha, Thorberg & Samios, 2012b; Lyvers, Mayer, Needham & Thorberg, 2019c; Lyvers, Hasking, Albrecht & Thorberg, 2012a; Lyvers, Makin, Toms, Thorberg & Samios, 2014c; Lyvers, Simons, Hayes & Thorberg, 2014b; Lyvers, Coundouris, Edwards & Thorberg, 2018b; Lyvers, Narayanan & Thorberg, 2019d; Lyvers, *et al*., 2019c; Patwardhan *et al*., 2019) and thirteen were based on student populations (Andres, Castanier & Le Scanff, 2014; Bauer & Ceballos 2014; Bladt, 2002; Bruce, Curren & Williams, 2012; Bujarski, Klanecky & McChargue, 2010; Gilan, Zakiei, Reshadat, Komasi & Ghasemi, 2015; Greene, Hasking & Boyes, 2020; Hahn, Simons & Simons, 2016. Lyvers *et al*., 2014d; Lyvers, Lysychka & Thorberg, 2014a; Lyvers, Hanigan & Thorberg 2018a, Lyvers, Mayer, Needham & Thorberg, 2019b, Shishido, Gaher & Simons, 2013).

The majority of the studies concerned alcohol use or alcohol disorders (n = 36) (Andres *et al*., 2014; Bauer & Ceballos 2014; Betka *et al*., 2018; Bladt, 2002; Bruce *et al*., 2012; Bujarski *et al*., 2010; Bulai & Enea, 2016; Chaudhury *et al*., 2006; Craparo, Ardino, Gori & Caretti, 2014; Founta, Adamzik, Tobin, Kirby & Hevey, 2019; Ghorbani, Khosravani, Sharifi Bastan & Jamaati Ardakani, 2017; Greene *et al*., 2020; Hahn *et al*., 2016; Hasking & Claes, 2020; Honkalampi *et al*., 2010; Knapton *et al*., 2018, Kopera, Trucco, Jakubczyk *et al*., 2018; Loas *et al*., 2000; Lyvers *et al*., 2012a, 2012b; Lyvers, *et al*., 2014a, 2014b, 2014c, 2018a, 2018b, 2019b, 2019c, 2019d; Lyvers, McCann, Coundouris, Edwards & Thorberg, 2018c; Lyvers *et al*., 2019c; Maurage, Grynberg, Noel *et al*., 2011; Pedersen, Sorensen, Bruun, Christensen & Vedsted, 2016; Shishido *et al*., 2013; Speranza *et al*., 2004; Zdankiewicz‐Ścigała & Ścigała, 2018; Zdankiewicz‐Ścigała & Ścigała, 2020) or both alcohol and drug use (n = 1) (Speranza *et al*., 2004). Seven studies included patients with drug use (heroin) (Craparo *et al*., 2016; Meziou *et al*., 2019; Parolin *et al*., 2017; Bashapoor, Hosseini‐Kiasari, Daneshvar & Kazemi‐Taskooh, 2015), cannabis use (Bulai & Enea, 2016), sedative use (Gilan *et al*., 2015) and methamphetamine use (Payer *et al*., 2011). A total of 28 studies included young participants with a mean age of 30 years or less and in 24 studies the proportion of males was over 50%. Some of the studies produced findings for the TAS‐20 total score only, some for the dimension only, and some for both the TAS‐20 total score and the dimension. Thus, the number of studies providing data for each of the outcomes varied between 28 and 46.

Almost half of the studies (n = 24) were from Europe, 10 studies were from Australia, five from USA, five from Asia, and two from Africa (Table 1). Most of the studies reported results for more than one alexithymia score, for example TAS‐20 total score and all three dimensions.

After the quality assessment of the eligible studies, we concluded that the overall quality of studies was poor or fair, mainly because of the cross‐sectional study design, which was one of the aspects increasing the risk of bias in the quality assessment tool (Table S2).

### Association between alexithymia and substance use

**Fig. 2:**
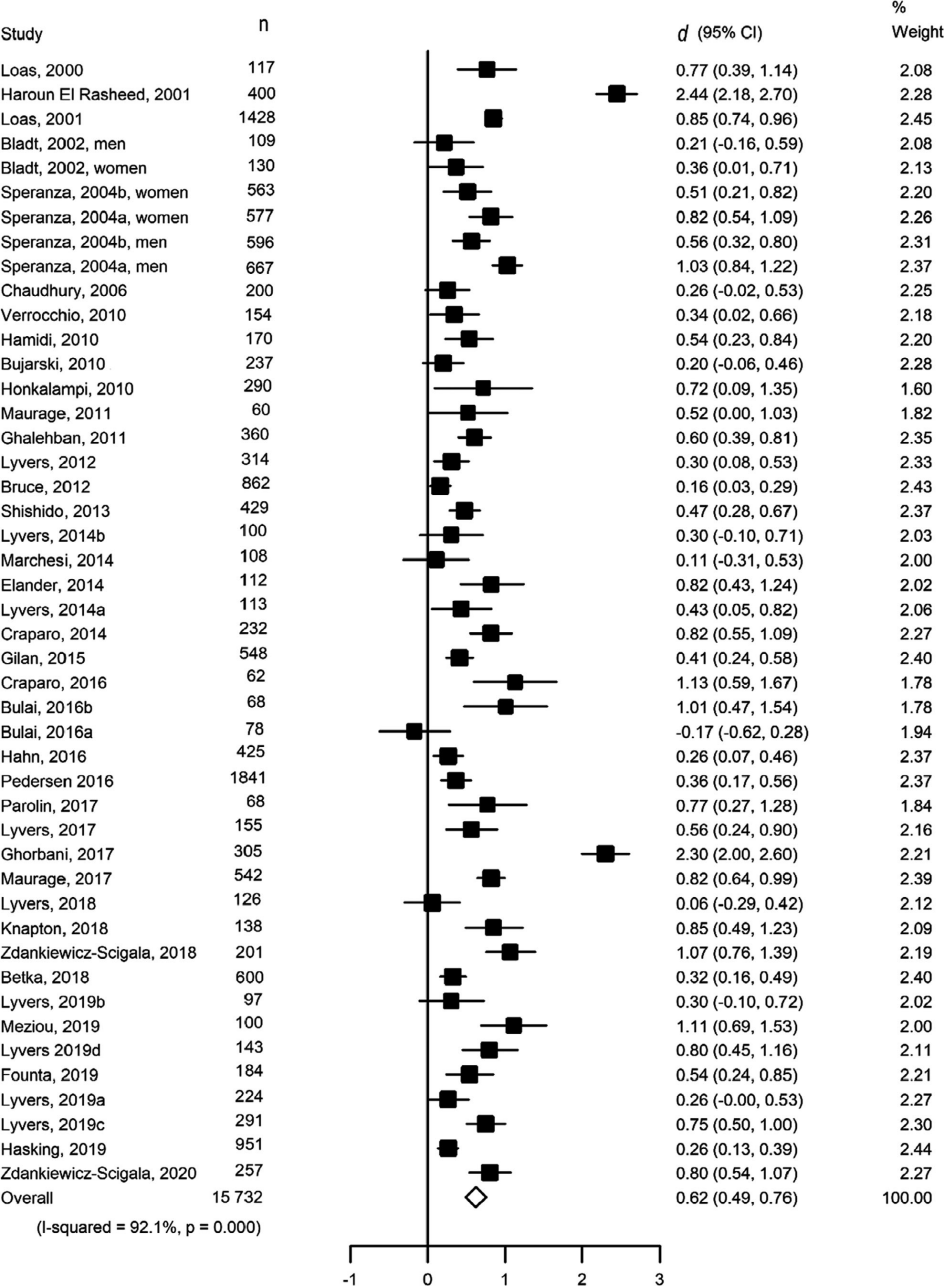
Association between alexithymia (TAS‐20 total score) and substance use.

There was a total of 46 estimates for the TAS‐20 total score. Meta‐analysis (Fig. 2) suggested a strong positive association between alexithymia and any substance use (*d* = 0.62, 95% CI 0.49–0.76), with high heterogeneity between studies (*I* ^*2*^ = 92.1%). Despite the high heterogeneity, all but one of the studies showed a positive association between alexithymia and substance use (Bulai & Enea, 2016). Two clinical studies suggested a very strong positive association and were seen as outliers (Ghorbani *et al*., 2017; Rasheed, 2001). Removing these two studies did not significantly alter the results (*d* = 0.54, 95% CI 0.44–0.63, *I* ^*2*^ = 81.4%).

**Fig. 3:**
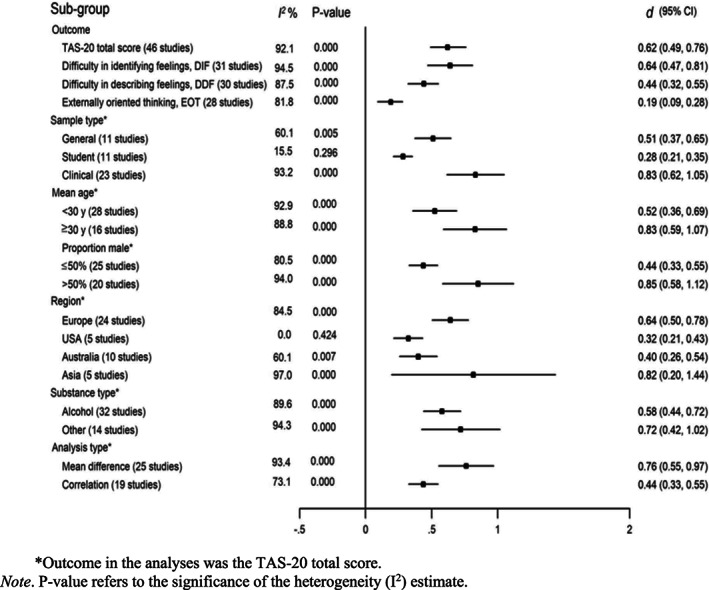
Sub‐group analyses of the association between alexithymia and substance use.

Results from the sub‐group analyses are presented in Fig. 3. Even within the sub‐groups, heterogeneity between studies was high. Only the 11 studies among student populations suggested no heterogeneity (*I* ^*2*^ = 15.5, P = 0.296), as well as the five studies carried out in the USA (*I* ^*2*^ = 0.0, P = 0.424) and the two studies with logistic regression as analysis type (*I* ^*2*^ = 10.9, P = 0.290).

With regard to the outcome type, the estimate for Difficulty in Identifying Feelings (DIF) was of the same magnitude (*d* = 0.64, 95% CI 0.47–0.81) as the TAS‐20 total score, whereas the estimates for Difficulty in Describing Feelings (DDF) and Externally Oriented Thinking (EOT) were lower in magnitude (*d* = 0.44, 95% CI 0.32–0.55 and *d* = 0.19, 95% CI 0.09–0.28), with a statistically significant interaction found in meta‐regression (*P* = 0.025).

There was also a significant interaction among sample types (*P* = 0.049). Clinical samples yielded higher estimates than student samples (*d* = 0.83, 95% CI 0.62–1.05 versus *d* = 0.28, 95% CI 0.21–0.35), whereas studies from general populations gave estimates between these two (d = 0.51, 95% CI 0.37–0.65).

There was no significant difference between studies regarding age of the participants (*P* = 0.051 for interaction in meta‐regression), but those with a higher proportion of male participants suggested a stronger association between alexithymia and substance use (*P* = 0.004; *d* = 0.44, 95% CI 0.33–0.55 when the proportion of men was ≤50% and *d* = 0.85, 95% CI 0.58–1.12 when the proportion of men was >50%). Other subgroup differences (region, substance type and analysis type) were small (*P* ‐values for interaction 0.130, 0.375 and 0.051, respectively).

Study‐specific forest plots for the association of alexithymia with alexithymia dimensions DIF, DDF and EOT are presented in Figs. [Link], [Link]. The funnel plot for the TAS‐20 total score results appears symmetric (Fig. S4), and there was no evidence of an association between study size and the estimates (Egger's test B = 1.07; *P* = 0.42), not even after exclusion of the two outlier estimates from the analysis (B = 0.40, *P* = 0.65).

## DISCUSSION

This meta‐analysis confirmed a strong association between alexithymia and substance use and identified some subgroup differences in the association. We identified 52 studies that provided estimates for the association between alexithymia and/or its dimension, and substance use measured by alcohol or illicit drug use. We found a strong positive association between alexithymia and any substance use (*d* = 0.62), and in the dimension the association appeared to be strongest for Difficulty in Identifying Feelings (DIF, *d* = 0.64), followed by Difficulty in Describing Feelings (DDF, *d* = 0.44), and the weakest association for Externally Oriented Thinking (EOT, *d* = 0.19). The associations for alcohol use and illicit drug use were similar, whereas the strongest association was found in studies with clinical patient populations. Alexithymia was also more strongly associated with substance use in studies with most male participants.

A previous systematic review (without meta‐analysis) (Pigoni *et al*., 2020) found a relationship between severity of alcohol use disorder and alexithymia among patients with alcohol use disorders. Another review (Thorberg, *et al*., 2009) summarized that alexithymia may have the potential to interfere with treatment outcomes among alcohol‐dependent patients. A previous meta‐analysis (Pigoni *et al*., 2020) showed that EOT was associated with risky drinking but not NNSI. We included all relevant study types which provided estimates for the association between alexithymia and any substance use and found, in line with previous research, that the association may be stronger among patients with severe alcohol use disorders than among the general population or students. The reasons for this finding may be related to bi‐directional impacts, that is, in addition to alexithymia increasing alcohol use, alcoholism involves a tendency to affect personality, including alexithymic traits, in a negative way (Kauhanen, Julkunen & Salonen, 1992). In addition, the association between alexithymia and substance use was stronger in older (≥ 30 years) participants, which is noted in an earlier study (Pigoni *et al*., 2020). Interestingly, a community‐based study showed, that affective symptoms are common in severe alcohol use disorders and it may promote further heavy drinking (Ehlers, Gilder, Gizer & Wilhelmsen, 2019). In addition, many studies have shown that probabilities of having an alcohol use disorder among those with a drug use disorder is significantly greater than among those without a drug use disorder and vice versa (Simons, Carey & Wills, 2009; Stinson, Grant, Dawson, Ruan, Huang & Saha, 2005). However, because almost all the studies were cross‐sectional, we could not test temporal order in associations to evaluate the direction of causality. Moreover, even though we found a strong association between alexithymia and the abuse of substances other than alcohol, it should be noted that due to insufficient data available for analyses we were unable to conduct individual analyses for substances other than alcohol. As different types of drugs have different impact on the central nervous system and consequently behavior, sedatives such as opioids, (i.e., heroin, buprenorphine) and stimulants (cocaine, methamphetamine), are likely to have different types of associations with alexithymic features. Therefore, our findings regarding the association between alexithymia and the use of substances other than alcohol should be approached with caution.

The reasons for the stronger association among male‐dominated studies are unknown but may reflect the fact that of the studies reporting results for the TAS‐20 total score, 77% of clinical studies were male‐dominated whereas the corresponding proportion of men in the general and student populations were 10% and 9%, respectively. There is a body of literature on male alexithymia, with a hypothesis of “normative male alexithymia” in the socialization process, which means that men have been discouraged from expressing their emotions during their development and this may have made them more vulnerable than women (Levant, Allen & Lien, 2019). In addition, Luminet, de Sousa Uva, Fantini and de Timary (2016) investigated a group of alcohol‐dependent patients and found a positive interaction between depression and gender in the prediction of craving for alcohol. For women, the link between depression and craving was associated with scoring higher on “difficulties describing feelings” but in men this link scored higher on “externally‐oriented thinking.” Further research is therefore needed to examine whether it is the clinical status that determines the association between alexithymia and substance use or whether there is actually a stronger association among men than women.

We found stronger associations of substance use with the emotion‐related dimensions (DIF and DFF) than with the cognition‐related dimension (EOT) of alexithymia. Several studies among patients with substance use disorder (Thorberg, *et al*., 2010) and psychiatric inpatients (Preece, Becerra, Robinson, Dandy & Allan, 2018) have found acceptable internal consistency, test–retest reliability and scale homogeneity for TAS‐20, but not in EOT scale. Similarly, a previous meta‐analysis based on 19 studies (Li, Zhang, Guo, Zhang 2015) revealed a positive association between depression and the TAS‐20 total score, DIF, and DDF, and a weak association was found between EOT and depressive symptoms. Previous longitudinal studies have shown that among recovering psychiatric patients, DIF and DDF scores decreased along with the alleviation of psychiatric symptoms, whereas EOT remained unchanged (Honkalampi, De Berardis, Vellante & Viinamäki, 2018; Rufer, Albrecht, Zaum *et al*., 2010; Saarijärvi, Salminen & Toikka, 2001). It is not clear why DIF and DDF are more closely related to mental and substance use disorders while the association with externally oriented thinking is weaker. Probably DIF and DDF may be more state‐dependent than EOT (Henry, Phillips, Maylor, Hosie, Milne & Meyer, 2006). People with high DIF scores have difficulties in identifying their feelings, that is, they often just feel somatic sensations and undifferentiated feelings of distress. Those with high levels of DDF, in turn, have difficulties in describing feelings, which makes their social interaction difficult as they are not able to verbalize (Luminet & Zamariola, 2018) their emotions to others and may not seek social support from other people. These characteristics may lead to escape‐avoidance coping strategies, such as high amount of alcohol use (Skrzynski & Creswell, 2020). A recent study among patients with substance use disorder (Taurino, Antonucci, Taurisano & Laera, 2021) showed that among all SUD sub‐groups (cocaine use disorder, opioid use disorder, alcohol use disorder (AUD)), patients with AUD, patients showed more dysfunctional defenses, all patients used a maladaptive/assimilation defense style, which is related to the DIF factor, and to a worse psychological functioning.

Among different regions, very strong association between alexithymia and substance use was found for studies from Asia (n = 5, d = 0.82, 95%CI 0.20–1.44), a strong association for studies from Europe (n = 24, d = 0.64, 95% < CI 0.50–0.78), and only a medium association for studies from Australia (n = 10, d = 0.40, 95%CI 0.26–0.54) and USA (n = 5, d = 0.32, 95%CI 0.21–0.43) (*p* = 0001). However, these sub‐group differences (region, substance type, analysis type) were imprecisely estimated and the meta‐regression analyses did not suggest significant interaction. Based on the present evidence, there is no clear evidence of differences in the association between alexithymia and substance use between different countries, when the outcome is alcohol or illicit drug use, or depending on the chosen analysis type in the study.

### Clinical implications

This meta‐analysis showed that alexithymia and especially its dimensions DIF and DDF have a strong positive association between any substance use. So far, there is some evidence that alexithymia worsens treatment outcomes in individuals with alcohol abuse or dependence. Higher levels of alexithymia have been found to be associated with a higher rate of relapse among alcoholics (Birt, Sandor, Vaida & Birt, 2008). In this study, the associations for alcohol use and illicit were strongest among studies with the patient populations and male participants. de Haan, van der Palen, Wijdeveld, Buitelaar and De Jong (2014) found in a three‐week follow‐up among patients with substance use disorders that the scores of the TAS‐20 may be partly related to an addiction as a temporary state but it can also be a stable trait. There is some evidence that the treatment outcome of substance use patients with alexithymia depends on treatment modality among individuals with addiction. For example, Morie, Nich, Hunkele, Potenza & Carroll (2015) showed that methadone‐maintained cocaine abusers with high alexithymia respond better to computerized Cognitive Behavioral Therapy for drug dependence than individuals with low alexithymia. This evidence shows that cognitive style, for example, their difficulty to describe feelings to a therapist, can be one of the underlying explanations regarding the differences in the ability to benefit from therapeutic treatment (Morie & Ridout, 2018).

### Strengths and limitations

This meta‐analysis examined alexithymia and substance use in a wide variety of studies. Other strengths of this review include examination of the associations for alexithymia sub‐scales as well as several predefined subgroups, in addition to the overall alexithymia score and total population. However, there are also important limitations that should be acknowledged. First, only two databases were used to select studies, but both PubMed and Web of Science databases are very compressive. However, in addition to that, we searched the reference lists of the eligible studies for additional records. Second, the quality of studies was rated only fair to poor, mainly due to the study design which was cross‐sectional in the majority of studies. Alexithymia was based on self‐reports in all studies, and in many studies the use of alcohol was also based on self‐report–although in clinical studies with patient populations alcohol use disorder was clinically verified. Self‐report of both the exposure and the outcome may involve common method bias, which artificially inflates associations. Furthermore, bias due to unmeasured confounding cannot be ruled out in observational studies. Due to these limitations, this meta‐analysis does not allow us to make causal conclusions regarding the relationships between alexithymia and substance use. Third, we found a high level of heterogeneity between studies although all but one of them suggested a positive association between alexithymia and substance use. Investigation of this issue in subgroups provided little additional information on the potential causes of heterogeneity; only sub‐group analyses restricted to student populations (n = 11), to those carried out in the USA (n = 5) and to investigations based on logistic regression analysis as a method (n = 2) did not suggest high heterogeneity. Many small meta‐analyses are not able to estimate heterogeneity with much precision; in fact, we may have little confidence in any estimate beyond the average effect size, thus any statistical analysis cannot change the limitations of small meta‐analyses (see von Hippel, 2015). Further studies are needed to examine what factors may underlie the observed heterogeneity. Furthermore, most studies were from high‐income countries, which limits the generalizability of the findings to low‐ or middle‐income countries.

## CONCLUSIONS

The findings of this systematic review and meta‐analysis suggest a strong association between alexithymia and substance use, and a very strong association among patients with alcohol use disorders. The association may be stronger for the emotion‐related dimensions than for the cognition‐related dimension of alexithymia. More longitudinal studies are needed to test the direction of causality between alexithymia and substance use.

MK was supported by the UK Medical Research Council (grant MRC S011676), the US National Institute on Aging (NIA) (grant R01AG056477), the Academy of Finland (311492), NordForsk (grant 70521, the Nordic Research Programme on Health and Welfare), and the Finnish Work Environment Fund (190424), outside the area of this study.

## Supporting information


**Figure S1.** Study‐specific associations between Difficulty in Identifying Feelings sub‐score of alexithymia and substance use.

**Figure S2.** Study‐specific associations between Difficulty in Describing Feelings sub‐score of alexithymia and substance use.

**Figure S3.** Study‐specific associations between Externally Oriented Thinking sub‐score of alexithymia and substance use.

**Figure S4.** The funnel plot for the TAS‐20 total score.

**Table S1.** Search terms for the search in PubMed.

**Table S2.** Quality assessment of the studies in meta‐analysis.

Supplementary material

## Data Availability

Data sharing is not applicable to this article as no new data were created or analyzed in this study.
