# Supplementary material for: Association between alexithymia and substance use: A systematic review and meta‐analysis
Source: Scand J Psychol. 2022 Apr 18;63(5):427–38. doi: 10.1111/sjop.12821 (PMC9790486; doi:10.1111/sjop.12821)

**Supplementary Figure 1.** Study-specific associations between Difficulty in Identifying Feelings sub-score of alexithymia and substance use


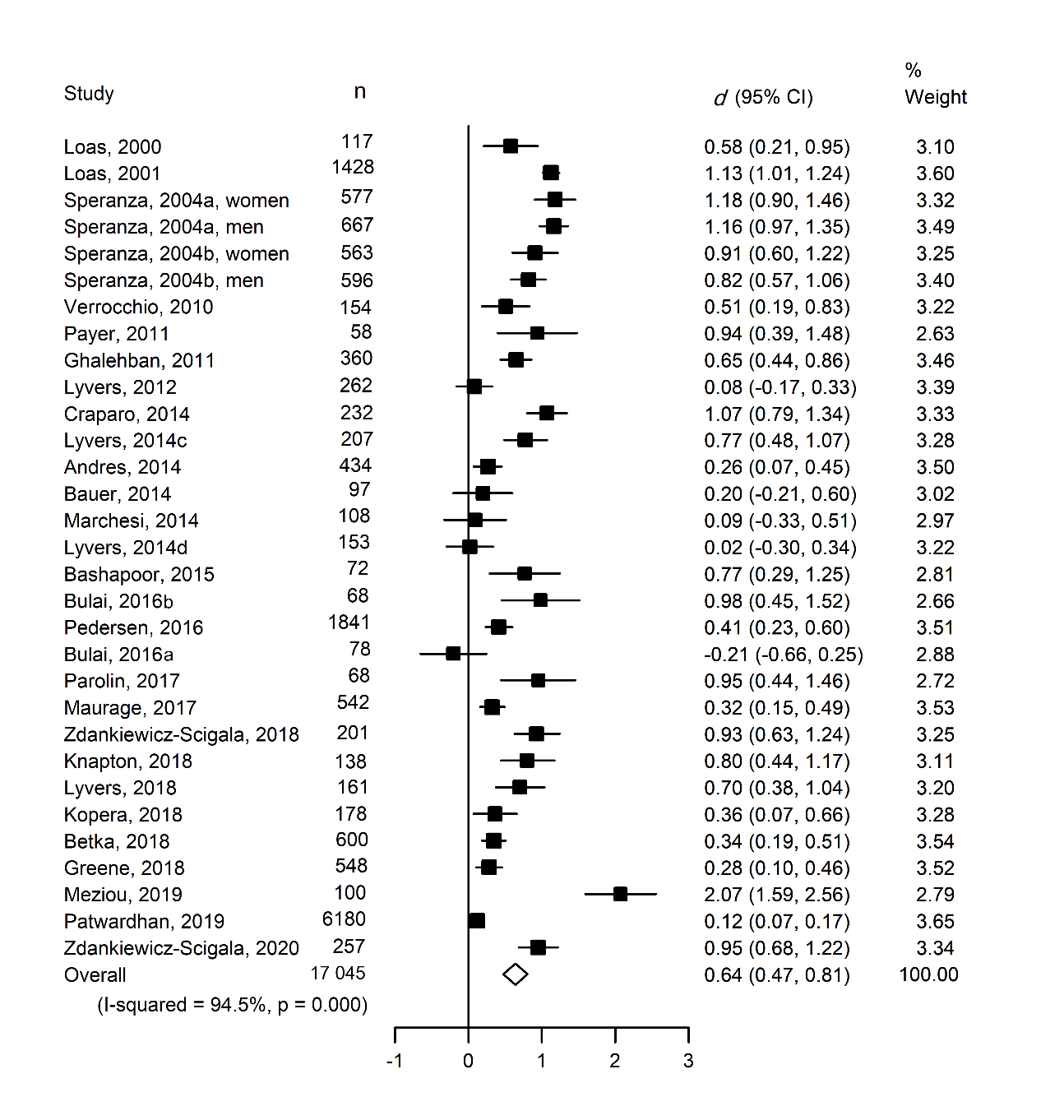

Supplement: Supplementary file 1 — Figure S1. Study‐specific associations between Difficulty in Identifying Feelings sub‐score of alexithymia and substance use. [file SJOP-63-427-s001.docx]
