# Supplementary material for: Association between alexithymia and substance use: A systematic review and meta‐analysis
Source: Scand J Psychol. 2022 Apr 18;63(5):427–38. doi: 10.1111/sjop.12821 (PMC9790486; doi:10.1111/sjop.12821)

**Supplementary Figure 2.** Study-specific associations between Difficulty in Describing Feelings sub-score of alexithymia and substance use


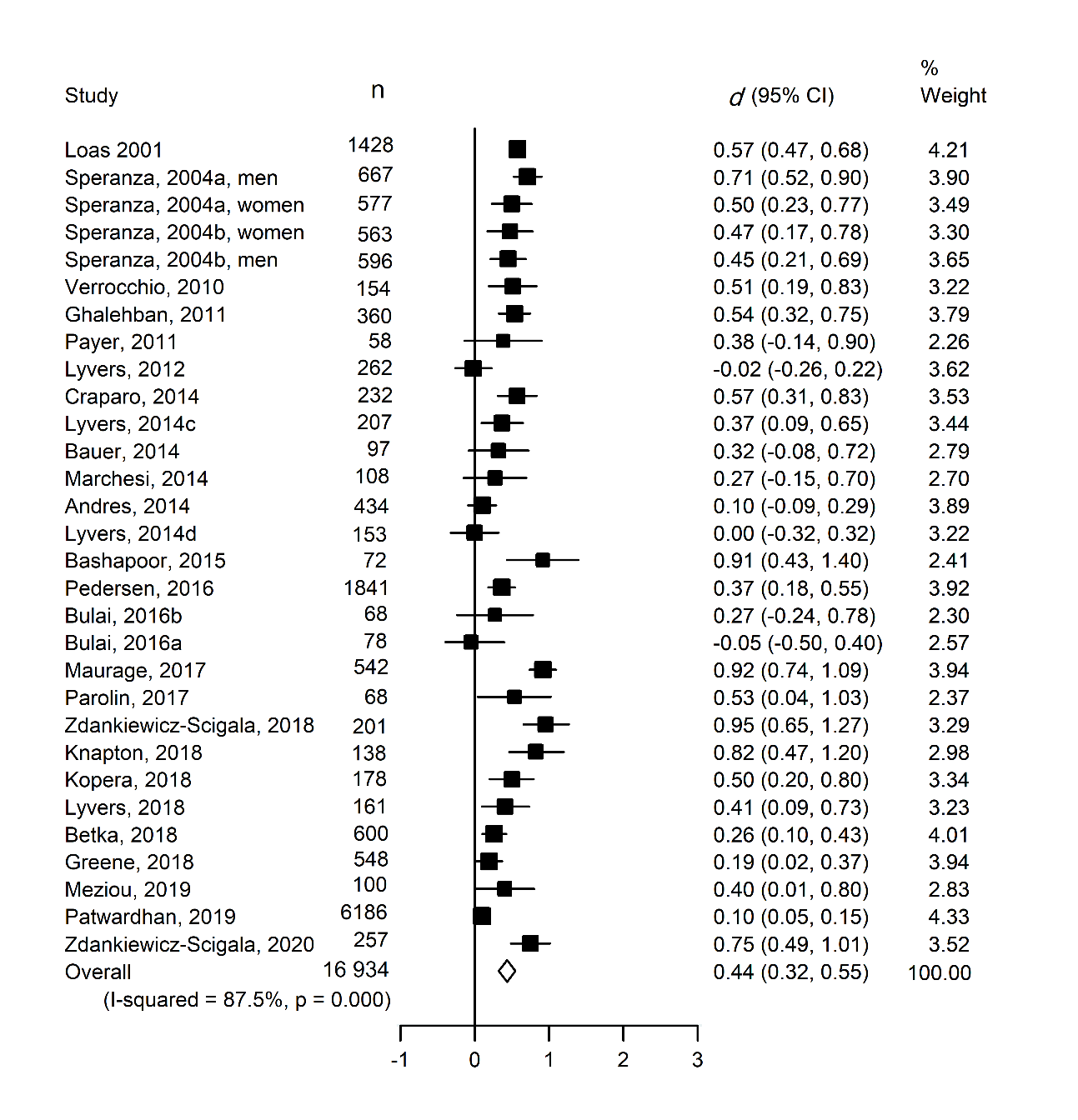

Supplement: Supplementary file 2 — Figure S2. Study‐specific associations between Difficulty in Describing Feelings sub‐score of alexithymia and substance use. [file SJOP-63-427-s004.docx]
