# Supplementary material for: Association between alexithymia and substance use: A systematic review and meta‐analysis
Source: Scand J Psychol. 2022 Apr 18;63(5):427–38. doi: 10.1111/sjop.12821 (PMC9790486; doi:10.1111/sjop.12821)

**Supplementary Figure 3.** Study-specific associations between Externally Oriented Thinking sub-score of alexithymia and substance use


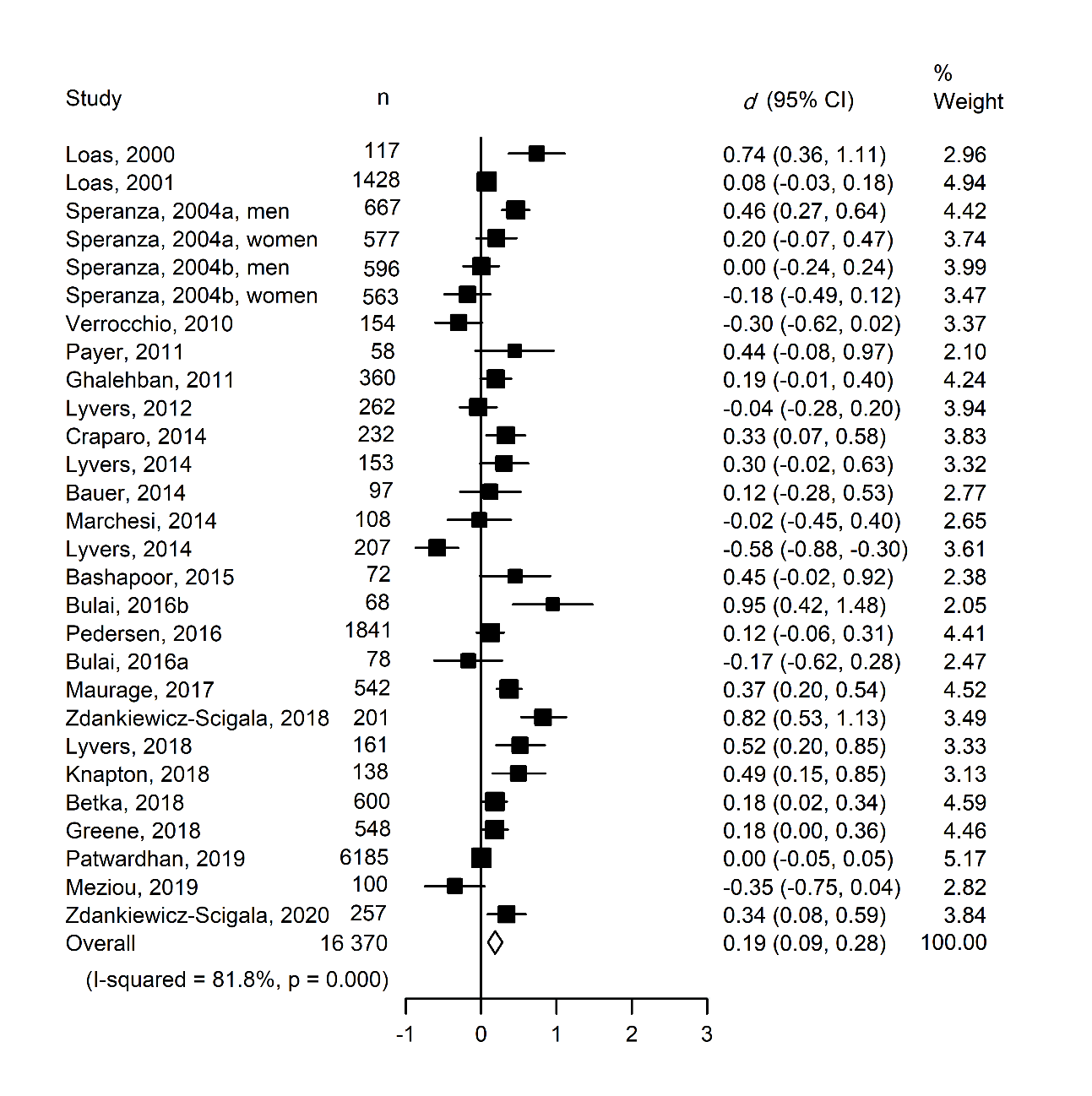

Supplement: Supplementary file 3 — Figure S3. Study‐specific associations between Externally Oriented Thinking sub‐score of alexithymia and substance use. [file SJOP-63-427-s006.docx]
