# Supplementary figures and images for: Association between alexithymia and substance use: A systematic review and meta‐analysis
Source: Scand J Psychol. 2022 Apr 18;63(5):427–38. doi: 10.1111/sjop.12821 (PMC9790486; doi:10.1111/sjop.12821)

**Supplement Figure 4**. The funnel plot for the TAS-20 total score


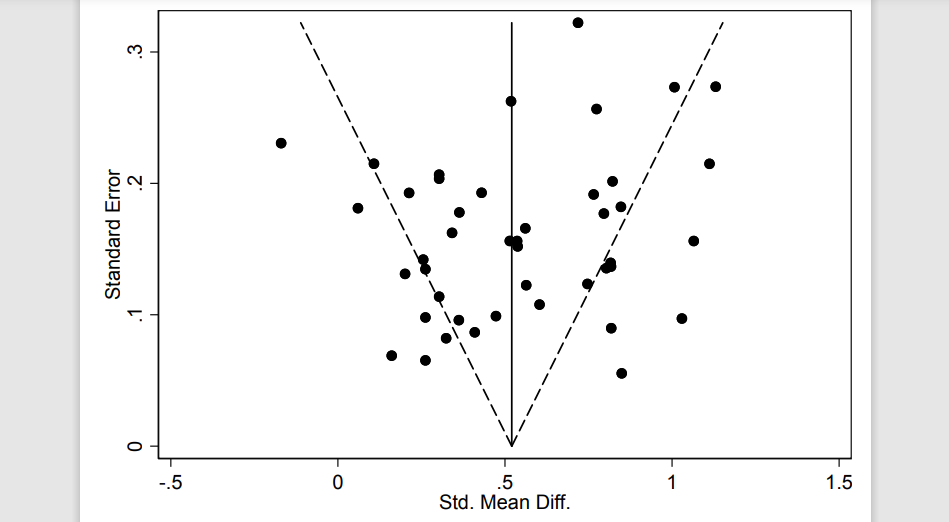

Supplement: Supplementary file 4 — Figure S4. The funnel plot for the TAS‐20 total score. [file SJOP-63-427-s002.docx]

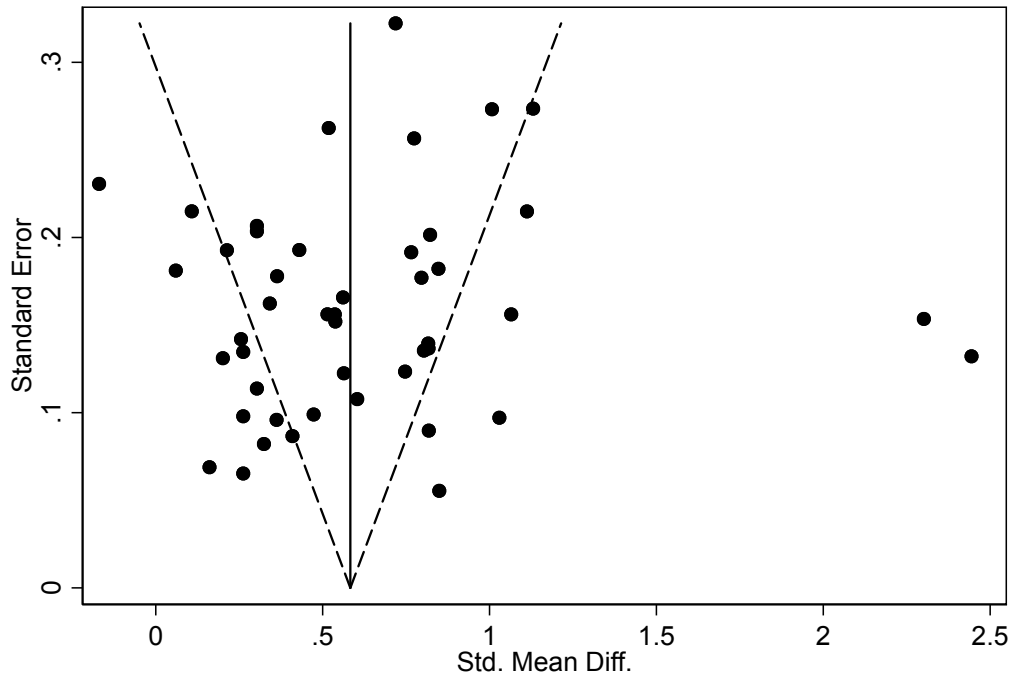

Supplement: Supplementary file 7 — Supplementary material [file SJOP-63-427-s003.pdf]
