# Supplementary material for: Association between alexithymia and substance use: A systematic review and meta‐analysis
Source: Scand J Psychol. 2022 Apr 18;63(5):427–38. doi: 10.1111/sjop.12821 (PMC9790486; doi:10.1111/sjop.12821)
Supplement: Supplementary file 5 — Table S1. Search terms for the search in PubMed. [file SJOP-63-427-s007.docx]

| **Supplementary Table 1.** Search terms for the search in PubMed |
| --- |
| #1,"Search ((Toronto) AND Alexithymia) AND Scale",1285  #2,"Search Alexithymia",24594  #3,"Search TAS-20",631  #4,"Search ((TAS-20) OR Alexithymia) OR (((Toronto) AND Alexithymia) AND Scale)",24604  #5,"Search addiction",73186  #6,"Search alcoholics",15002  #7,"Search (alcohol) AND abuse",118990,  #8,"Search (alcohol) AND intoxication",15792  #9,"Search (alcohol) AND use",94055,  #10,"Search ((alcohol) AND use) AND disorder",31550  #11,"Search (alcohol) AND drinking",85035  #12,"Search (((amphetamine-type) AND substance) AND use) AND disorder",1706  #13,"Search ((amphetamine) AND related) AND disorders",9430,  #14,"Search ((cannabis) AND use) AND disorder",1481  #15,"Search ((cocaine) AND use) AND disorder",1698  #16,"Search ((cocaine) AND related) AND disorder",4406  #17,"Search (drinking) AND behaviour",88754  #18,"Search (drug) AND use",419684  #19,"Search ((inhalant) AND use) AND disorder",1830  #20,"Search (inhalant) AND abuse",1782  #21,"Search (marijuana) AND abuse",16953,  #22,"Search (marijuana) AND use",9923  #23,"Search ((opioid) AND related) AND disorders",24631  #24,"Search ((opioid) AND use) AND disorders",8697  #25,"Search ((stimulant) AND use) AND disorder",2431  #26,"Search (substance) AND abuse",159647  #27,"Search (substance) AND use",60053,06  #28,"Search ((substance) AND related) AND disorders",108211  #29,"Search (((((((((((((((((((((((addiction) OR alcoholics) OR ((alcohol) AND abuse)) OR ((alcohol) AND intoxication)) OR ((alcohol) AND use)) OR (((alcohol) AND use) AND disorder)) OR ((alcohol) AND drinking)) OR ((((amphetamine-type) AND substance) AND use) AND disorder)) OR (((amphetamine) AND related) AND disorders)) OR (((cannabis) AND use) AND disorder)) OR (((cocaine) AND use) AND disorder)) OR (((cocaine) AND related) AND disorder)) OR ((drinking) AND behaviour)) OR ((drug) AND use)) OR (((inhalant) AND use) AND disorder)) OR ((inhalant) AND abuse)) OR ((marijuana) AND abuse)) OR ((marijuana) AND use)) OR (((opioid) AND related) AND disorders)) OR (((opioid) AND use) AND disorders)) OR (((stimulant) AND use) AND disorder)) OR ((substance) AND abuse)) OR ((substance) AND use)) OR (((substance) AND related) AND disorders)",769267  #30,"Search (((((((((((((((((((((((((addiction) OR alcoholics) OR ((alcohol) AND abuse)) OR ((alcohol) AND intoxication)) OR ((alcohol) AND use)) OR (((alcohol) AND use) AND disorder)) OR ((alcohol) AND drinking)) OR ((((amphetamine-type) AND substance) AND use) AND disorder)) OR (((amphetamine) AND related) AND disorders)) OR (((cannabis) AND use) AND disorder)) OR (((cocaine) AND use) AND disorder)) OR (((cocaine) AND related) AND disorder)) OR ((drinking) AND behaviour)) OR ((drug) AND use)) OR (((inhalant) AND use) AND disorder)) OR ((inhalant) AND abuse)) OR ((marijuana) AND abuse)) OR ((marijuana) AND use)) OR (((opioid) AND related) AND disorders)) OR (((opioid) AND use) AND disorders)) OR (((stimulant) AND use) AND disorder)) OR ((substance) AND abuse)) OR ((substance) AND use)) OR (((substance) AND related) AND disorders))) AND (((TAS-20) OR Alexithymia) OR (((Toronto) AND Alexithymia) AND Scale))",2539,06  #31,"Search (""2000/01/01""[Date - Publication] : ""3000""[Date - Publication])",16653318  #32,"Search ((""2000/01/01""[Date - Publication] : ""3000""[Date - Publication])) AND ((((((((((((((((((((((((((addiction) OR alcoholics) OR ((alcohol) AND abuse)) OR ((alcohol) AND intoxication)) OR ((alcohol) AND use)) OR (((alcohol) AND use) AND disorder)) OR ((alcohol) AND drinking)) OR ((((amphetamine-type) AND substance) AND use) AND disorder)) OR (((amphetamine) AND related) AND disorders)) OR (((cannabis) AND use) AND disorder)) OR (((cocaine) AND use) AND disorder)) OR (((cocaine) AND related) AND disorder)) OR ((drinking) AND behaviour)) OR ((drug) AND use)) OR (((inhalant) AND use) AND disorder)) OR ((inhalant) AND abuse)) OR ((marijuana) AND abuse)) OR ((marijuana) AND use)) OR (((opioid) AND related) AND disorders)) OR (((opioid) AND use) AND disorders)) OR (((stimulant) AND use) AND disorder)) OR ((substance) AND abuse)) OR ((substance) AND use)) OR (((substance) AND related) AND disorders))) AND (((TAS-20) OR Alexithymia) OR (((Toronto) AND Alexithymia) AND Scale)))",1767  #33,"Search English[Language]",25457684  #34,"Search ((((""2000/01/01""[Date - Publication] : ""3000""[Date - Publication])) AND ((((((((((((((((((((((((((addiction) OR alcoholics) OR ((alcohol) AND abuse)) OR ((alcohol) AND intoxication)) OR ((alcohol) AND use)) OR (((alcohol) AND use) AND disorder)) OR ((alcohol) AND drinking)) OR ((((amphetamine-type) AND substance) AND use) AND disorder)) OR (((amphetamine) AND related) AND disorders)) OR (((cannabis) AND use) AND disorder)) OR (((cocaine) AND use) AND disorder)) OR (((cocaine) AND related) AND disorder)) OR ((drinking) AND behaviour)) OR ((drug) AND use)) OR (((inhalant) AND use) AND disorder)) OR ((inhalant) AND abuse)) OR ((marijuana) AND abuse)) OR ((marijuana) AND use)) OR (((opioid) AND related) AND disorders)) OR (((opioid) AND use) AND disorders)) OR (((stimulant) AND use) AND disorder)) OR ((substance) AND abuse)) OR ((substance) AND use)) OR (((substance) AND related) AND disorders))) AND (((TAS-20) OR Alexithymia) OR (((Toronto) AND Alexithymia) AND Scale))))) AND English[Language]",1637, |
