# Supplementary material for: Association between alexithymia and substance use: A systematic review and meta‐analysis
Source: Scand J Psychol. 2022 Apr 18;63(5):427–38. doi: 10.1111/sjop.12821 (PMC9790486; doi:10.1111/sjop.12821)
Supplement: Supplementary file 6 — Table S2. Quality assessment of the studies in meta‐analysis. [file SJOP-63-427-s005.docx]

**Supplementary Table 2.** Quality assessment of the studies in meta-analysis

| 1. **Quality assessment for cross-sectional and cohort studies** | | | | | | |  |  |  |  |  |  |  |  |  |  |
| --- | --- | --- | --- | --- | --- | --- | --- | --- | --- | --- | --- | --- | --- | --- | --- | --- |
| Study | 1. Was the research question or objective in this paper clearly stated? | 2. Was the study po-pulation clearly specified and defined? | 3. Was the partici-pation rate of eligible persons at least 50%? | 4. Were all the subjects selected or recruited from the same or similar populations (including the same time period)? Were inclusion and exclusion criteria for being in the study prespecified and applied uniformly to all participants? | 5. Was a sample size justification, power de-scription, or variance and effect estimates provided? | 6. For the analyses in this paper, were the exposure(s) of interest measured prior to the outcome(s) being measured? | 7. Was the timeframe sufficient so that one could reasonably expect to see an association between exposure and outcome if it existed? | 8. For exposures that can vary in amount or level, did the study examine different levels of the exposure as related to the outcome (e.g., categories of exposure, or exposure measured as continuous variable)? | 9. Were the exposure measures (independent variables) clearly defined, valid, reliable, and implemented consistently across all study participants? | 10. Was the ex-posure(s) assessed more than once over time? | 11. Were the outcome measures (dependent variables) clearly defined, valid, reliable, and implemented consistently across all study participants? | 12. Were the outcome assessors blinded to the exposure status of participants? | 13. Was loss to follow-up after baseline 20% or less? | 14. Were key potential confounding variables measured and adjusted statistically for their impact on the relationship between exposure(s) and outcome(s)? | Number with NO/NR | TOTAL RATING* |
| Andres et al 2014 | yes | yes | NR | yes | no | no | NA | yes | yes | no | yes | no | NA | no | 5 | POOR |
| Betka et al. 2018 | yes | yes | NR | yes | no | no | NA | yes | yes | no | yes | no | NA | no | 6 | POOR |
| Bauer et al. 2014 | yes | yes | NR | yes | ni | no | NA | yes | yes | no | yes | no | NA | no | 6 | POOR |
| Bladt 2002 | yes | yes | yes | yes | no | no | NA | yes | yes | no | yes | no | NA | yes | 4 | POOR |
| Bruce et al., 2012 | yes | yes | NR | no | no | no | NA | yes | yes | no | yes | no | NA | no | 6 | POOR |
| Bujarski et al., 2010 | yes | yes | NR | yes | no | no | NA | yes | yes | no | yes | no | NA | no | 6 | POOR |
| Elander, J. et al., 2014 | yes | yes | NR | yes | no | no | NA | yes | yes | no | yes | no | NA | no | 6 | POOR |
| Founta et al., 2019 | yes | yes | yes | yes | no | no | NA | yes | yes | no | yes | no | NA | no | 5 | POOR |
| Gilan et al., 2015 | yes | yes | NR | yes | no | no | NA | yes | yes | no | yes | no | NA | no | 6 | POOR |
| Greene et al., 2019 | yes | yes | NR | yes | no | no | NA | yes | yes | no | yes | no | NA | no | 6 | POOR |
| Hahn et al., 2016 | yes | yes | NR | yes | no | no | NA | yes | yes | no | yes | no | NA | no | 6 | POOR |
| Hasking & Claes 2020 | yes | yes | NR | yes | no | no | NA | yes | yes | no | yes | no | NA | yes | 5 | POOR |
| Honkalampi et al., 2010 | yes | yes | yes | yes | no | yes | yes | yes | yes | no | yes | yes | yes | no | 3 | FAIR |
| Knapton et al., 2018 | yes | yes | NR | yes | no | no | NA | yes | yes | no | yes | no | NA | no | 6 | POOR |
| Lyvers et al. 2019a | yes | yes | NR | yes | no | no | NA | yes | yes | no | yes | no | NA | no | 6 | POOR |
| Lyvers et al. 2018b | yes | yes | NR | yes | no | no | NA | yes | yes | no | yes | no | NA | no | 6 | POOR |
| Lyvers et al. 2018a | yes | yes | NR | yes | no | no | NA | yes | yes | no | yes | no | NA | no | 6 | POOR |
| Lyvers et al. 2012a | yes | yes | NR | yes | no | no | NA | yes | yes | no | yes | no | NA | no | 6 | POOR |
| Lyvers et al. 2019b | yes | yes | NR | yes | no | no | NA | yes | yes | no | no | no | NA | no | 6 | POOR |
| Lyvers et al. 2014a | yes | yes | NR | yes | no | no | NA | yes | yes | no | yes | no | NA | no | 6 | POOR |
| Lyvers et al. 2014d | yes | yes | NR | yes | no | no | NA | yes | yes | no | yes | no | NA | no | 6 | POOR |
| Lyvers et al. 2019c | yes | yes | NR | yes | no | no | NA | yes | yes | no | yes | no | NA | no | 6 | POOR |
| Lyvers et al. 2018c | yes | yes | NR | yes | no | no | NA | yes | yes | no | yes | no | NA | no | 6 | POOR |
| Lyvers et al. 2019d | yes | yes | NR | yes | no | no | NA | yes | yes | no | yes | no | NA | yes | 5 | POOR |
| Lyvers et al. 2012b | yes | yes | NR | yes | no | no | NA | yes | yes | no | yes | no | NA | no | 6 | POOR |
| Lyvers et al. 2014b | yes | yes | NR | yes | no | no | NA | yes | yes | no | yes | no | NA | no | 6 | POOR |
| Patwardhan et al. 2019 | yes | yes | yes | yes | no | yes | yes | yes | yes | no | yes | yes | no | no | 4 | FAIR |
| Pedersen et al., 2016 | yes | yes | no | yes | no | no | NA | yes | yes | no | yes | no | NA | yes | 5 | POOR |
| Shishido et al., 2013 | yes | yes | NR | yes | no | no | NA | yes | yes | no | yes | no | NA | no | 6 | POOR |
| Zdankiewicz-Ścigała & Ścigała, 2020 | yes | yes | NR | yes | no | no | NA | yes | yes | no | yes | no | NA | no | 6 | POOR |

*Note. The weight of each point in the assessment is not equal (e.g. “no” in Point 6 denotes high level of bias and thus poor quality).
